# The Poncho Lamina Technique: A Protocol for Hard and Soft Tissue Augmentation in Atrophic Ridges Receiving Adjacent Implants

**DOI:** 10.3390/medicina59111994

**Published:** 2023-11-13

**Authors:** Alexander Tzovairis, Marius Leretter, Bart Vandenberghe, Roberto Rossi

**Affiliations:** 1 Private Practice, 1040 Brussels, Belgium; 2 Department of Prosthodontics, Faculty of Dental Medicine, Victor Babes University of Medicine and Pharmacy, 300041 Timisoara, Romania; mariusleretter@yahoo.com; 3 Advimago, Center for Advanced Oral Imaging, 1050 Brussels, Belgium; bart.vandenberghe@advimago.be; 4 Private Practice, 16121 Genova, Italy

**Keywords:** bone augmentation, cervical profile, cortical lamina, customized abutments, dental implants

## Abstract

The current scientific knowledge and guidelines in bone and soft tissue augmentation suggest the use of staged surgical workflows as the gold standard of regenerative procedures during implant therapy. In this context, the process is always the same, regardless of the techniques applied: an alternate series of surgical acts that follow one another after the completion of a specific period of osseointegration or graft maturation. As a result, the overall surgical treatment is often long and invasive and induces scar tissue formation. This article proposes a novel, fast, and less-invasive biphasic protocol with the use of a well-documented cortical barrier mounted on healing screws that are further replaced by customized abutments at an early second stage. Two cases are reported, one for an upper maxillary edentulous area and the other for a mandibular, with a total of four implants placed. The results at 4 months postop showed an optimal soft tissue configuration for both cases, with adequate cervical profile generation and a sufficient supracrestal complex height above the implant platforms. Significant bone gains were also recorded through CBCT data collection, either with alveolar width measurements on axial slices, the superposition of pre-op and post-op datasets, or 3D visualization after bone volume segmentation.

## 1. Introduction

Bone regeneration and augmentation in dental implantology constitute a specific area of interest for both researchers and clinicians. The basic elements that comprise its foundations have been well defined in the past and currently provide guidelines for success in the predominant clinical practice [1,2,3]. Single-bone augmentation has represented a great matter of interest for many years in oral and maxillofacial surgery, with the use of biomaterials or autologous bone. However, only recently has the importance of soft tissue augmentation and optimization been added into the whole process [4]. Keratinized tissue preservation or promotion and cervical profile generation are now accepted by the scientific community as key aspects of success in regenerative surgery [5,6,7,8,9,10]. As a consequence, a novel “Hybrid” hard-and-soft-tissue-orientated surgical augmentation approach is considered to be the best treatment strategy for stable results long lasting stable results [11,12]. Nevertheless, the current regenerative protocols support an analytical-staged workflow where each surgical act is followed by another after a specific time period, which refers either to implant osseointegration or to hard/soft tissue grafting material maturation. Under such circumstances, patients have to undergo long treatments that, on many occasions, take up to 6 or 9 months to reach the impression stage [13]. Therefore, several dental implant and biomaterial companies have introduced novel non-absorbable or resorbable products that promise to reduce the duration of surgical treatments (from bone regeneration to implant placement and soft tissue augmentation) to shorter periods and below the 6-month threshold [14]. Regardless of these efforts, such products seem to fail to diminish the number of surgical invasive interventions that often result in post-operative complications, pain, and scar tissue formation.

This case report features a new accelerated protocol for both hard and soft tissue augmentation with simultaneous implant placement, using a cortical barrier mounted on healing abutments, and proposes a faster, less invasive surgical workflow based on smart tissue manipulation.

## 2. Materials and Methods

The present case report includes 2 cases, one for the upper maxilla and one mandibular.

The inclusion criteria for the enrolment of the 2 patients were:

A. Patients seeking implant treatment and above 18 years of age;
B. Patients who signed an informed consent document and agreed to comply with the post-operative instructions and the follow-up planning;
C. Patients with good oral hygiene and no periodontal disease (plaque scores <25%, no pockets during probing, BoP levels <25%);
D. Patients with keratinized mucosa with a minimum width of 4 mm on the edentulous sites to be treated.

The exclusion criteria were:

A. Patients with pathologies that affect bone metabolism and wound healing;
B. Pregnancy and/or lactation during the treatment and healing period;
C. Smokers.

### 2.1. Case 1

**Figure 1:**
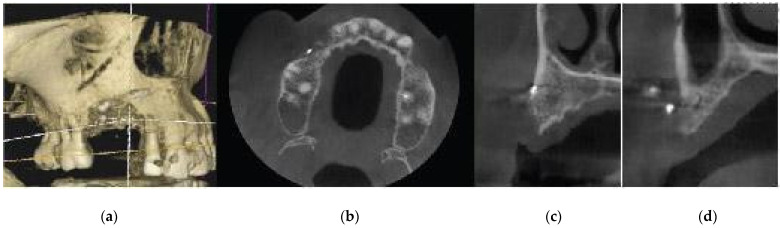
Pre-op CBCT situation—(**a**) 3D representation of the defect, (**b**) axial upper maxilla image, (**c**) cross-sectional image site 13, (**d**) cross-sectional image site 14.

**Figure 2:**
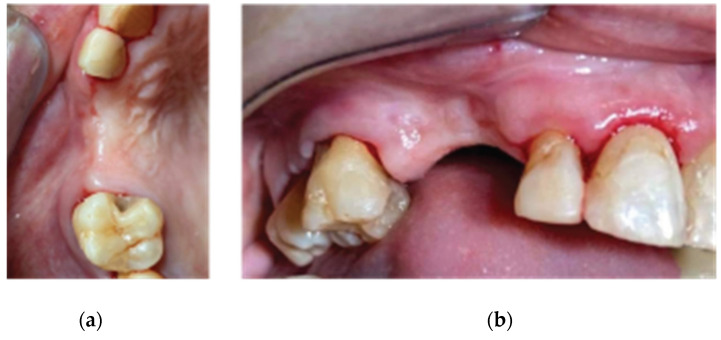
Pre-op clinical situation—(**a**) occlusal view, (**b**) lateral view.

A 72-year-old female patient attended our clinic seeking rehabilitation treatment on the upper edentulous 13–14 area. The patient was a healthy non-smoker without periodontal disease. A pre-op cone beam computed tomography (CBCT) scan was performed on the upper second quadrant. The CBCT results revealed an important 3-dimensional defect on area 13–14, with horizontal and vertical bone loss. The bone density was qualified as type 4 [15], and the distance from the most coronal edge of the ridge to the sinus floor was sufficient to place dental implants (13 mm for site 13 and 7.3 mm for site 14). Residual endodontic filling material adjacent to the bone structure was identified at both site 13 and site 14 (Figure 1). The clinical examination of the site did not reveal any periodontal pockets or mobility on neighboring teeth and, therefore, tooth elements 12 and 16 were both preserved and received an ultrasound scaling prior to surgical treatment (Figure 2).

The patient was treated with the Poncho Lamina accelerated protocol for multiple adjacent implants both at site 13 and at site 14. For the implant treatment itself, we selected and used Straumann SLA BLT Implants (Straumann Group, Basel, Switzerland). Bone regeneration was performed with Genos porcine particulated graft (Osteobiol by Tecnoss, Turin, Italy) and a well-documented Curved Lamina barrier 35 × 35 (Osteobiol by Tecnoss, Turin, Italy) [16,17]. Soft tissue optimization was performed with the use of the VPI Cervico System (VP Innovato Holdings LTD, Limassol, Cyprus) for anatomical abutment fabrication and cervical profile generation [18].

The surgical workflow consisted of a 2-stage process:

1. An initial stage for bone augmentation with simultaneous implant placement and emergence profile generation with the use of healing abutments (duration: from the beginning of treatment to complete implant osseointegration);
2. A second stage for soft tissue management and cervical profile generation with the use of anatomical abutments (duration: from implant osseointegration to complete graft maturation).

### 2.2. Stage 1

**Figure 3:**
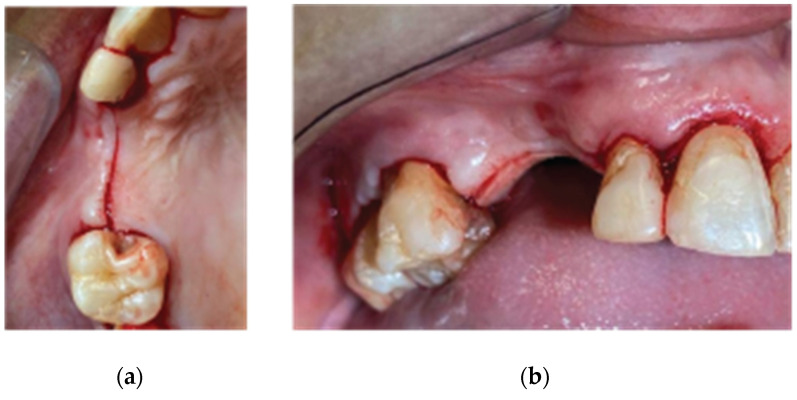
Incision design—(**a**) occlusal view, (**b**) lateral view.

**Figure 4:**
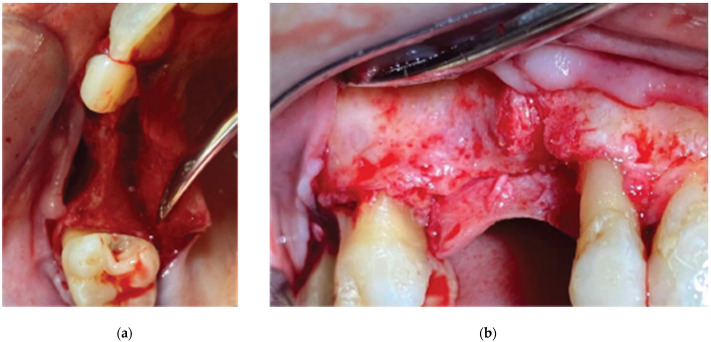
3D defect after flap elevation—(**a**) occlusal view, (**b**) lateral view.

Following the administering of local anesthesia (Articaine Hydrochloride/Adrenaline), access to the edentulous ridge was provided with a 15C blade, using intrasulcular buccal and palatal incisions from the distal area of tooth 17 to the distal aspect of tooth 21 and a mid-crestal incision on edentulous sites 13–14 (Figure 3). The interdental papilla areas were carefully dissected in split thickness, and 2 flaps (buccal–palatal) were meticulously elevated (Figure 4). The buccal flap was further released with a periosteal incision using a 15 blade and a periosteal elevator.

**Figure 5:**
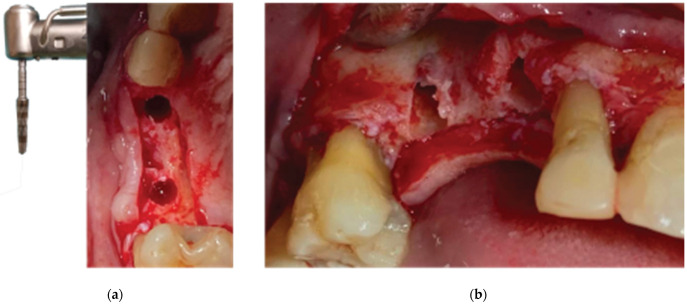
Ridge expansion and Protocol 1 crestal sinus lift with Versah drills—(**a**) occlusal view, (**b**) lateral view.

**Figure 6:**
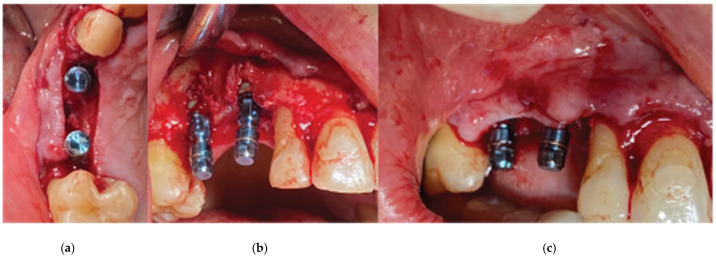
Straumann SLA BLT implant subcrestal placement—(**a**) occlusal view, (**b**) lateral view, (**c**) checking the buccal flap passivity prior to bone grafting.

An alveolar expansion protocol using Densah drills (Versah LLC, Jackson, MI, USA) followed at site 13, and a crestal Versah protocol 1 sinus lift was performed for site 14 (Figure 5) at an 800 RPM speed and 50 N torque in counterclockwise mode and under external irrigation [19]. A collagen sponge was carefully pushed through the site 14 osteotomy, and 2 Straumann 4.1-diameter SLA BLT implants were inserted (10 mm length for site 13, and 8 mm for site 14) subcrestally, with a final torque of 35 N.cm (Figure 6).

**Figure 7:**
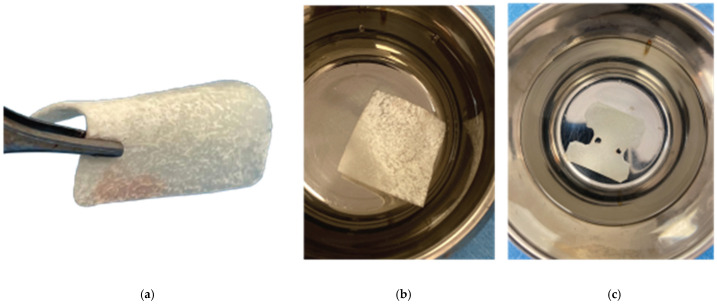
Curved Cortical Lamina 35 × 35 preparation. (**a**) Barrier not fully hydrated, (**b**) Barrier after hydration in saline, (**c**) Barrier with openings trimmed according to site topography.

After the site preparation and implant placement were completed, a curved cortical Lamina 35 × 35 mm (Osteobiol by Tecnoss, Turin, Italy) was hydrated in sterile saline water for 5 min. Following the hydration, 2 openings were trimmed out, using a rubber dam punch instrument. The locations of the openings referred to the exact locations of the implants at the surgical site, and they were further widened and refined with a micro-blade (SM67 Swann Morton). The cortical lamina was, finally, shaped with a 15 blade and scissors, according to site topography, and 2 Straumann healing abutments of 5 mm diameter and 4 mm height were positioned and secured through its openings (Figure 7).

**Figure 8:**
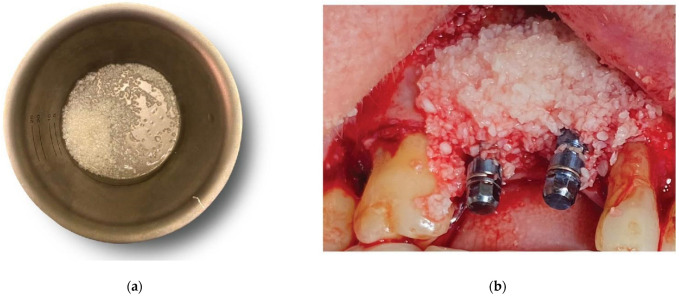
Overgrafting with Genos Xenograft - (**a**) Genos granules hydrated with saline, (**b**) grafting material in situ.

Prior to the barrier–abutment compound preparation, the site was overgrafted with 2 g of Genos 0.5 g (particle size: 0.5–1 mm—Osteobiol by Tecnoss, Turin, Italy) previously hydrated with sterile saline (Figure 8).

**Figure 9:**
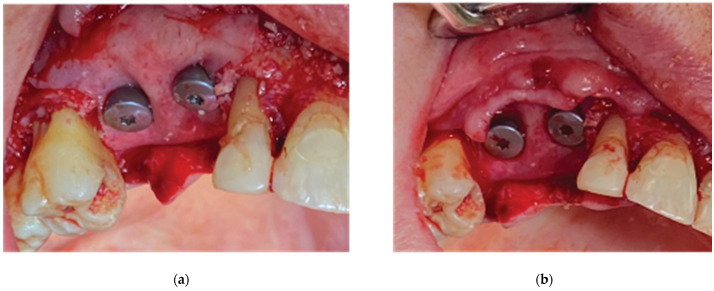
Healing abutment–Lamina compound in situ - (**a**) clinical situation after securing the healing abutments, (**b**) checking the passivity of the buccal flap over the compound.

After the bone graft was set on the buccal and palatal sides, care was taken to remove any granules remaining over the implant platforms. Then, the 2 flaps were gently pulled apart to enable the healing abutment–cortical-membrane compound placement, and the abutments were secured in place with a torque of 20 N.cm (Figure 9).

**Figure 10:**
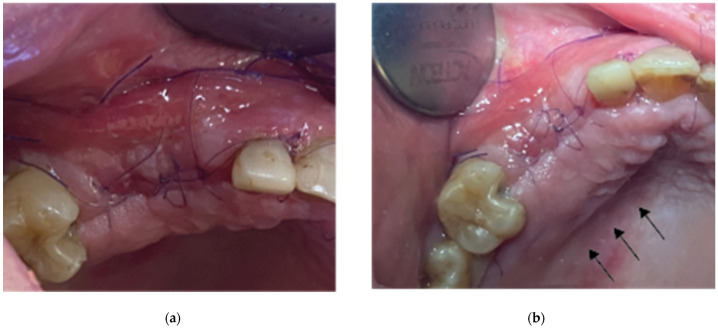
Clinical situation 2 weeks post op: (**a**) lateral view with primary closure with the 2 layers of sutures, (**b**) occlusal view with arrows revealing the palatal incision location.

Flap closure over the barrier and the abutments was performed with a palatal release incision and 2 layers of sutures, as follows (Figure 10):

- A palatal incision parallel to the alveolar ridge and located apically nearly 15 mm from the marginal palatal soft tissue, leaving the periosteum intact, was performed prior to the placement of sutures. Then, a split-thickness preparation below the palatal flap was performed until the dissected area reached the incision limits. After confirmation of the communication between both entries, the palatal flap coronal advancement was possible, ensuring a primary closure.
- An intermediate layer of horizontal mattress monofilament absorbable Glycolon 4.0 adaptation sutures (Resorba, Nuremberg, Germany) was placed below the muco-gingival line to secure an additional stabilization of the cortical barrier on both the buccal and the palatal side and the coronal advancement of both flaps.
- A superficial layer of vertical mattress (placed at the papillary areas between the teeth adjacent to the augmentation site), along with simple interrupted Glycolon 5.0 (Resorba, Nuremberg, Germany) closure sutures (placed at the inter-proximal abutment areas), provided primary flap closure and papilla stabilization on their initial pre-operative position.
- A superficial incision, performed with a 15 blade deep in the vestibule, was finally used to reduce muscle pulling during the post-operative healing period.

Following a 2–3 min gentle compression over the palatal incision with a sterile gauze to assure hemostasis, post-surgical instructions were given to the patient. She was advised to rinse with a disinfectant solution (BlueM Mouthwash, BlueM Oral care) 24 h after the surgery and twice a day for a period of 1 month [20]. The patient was also told to avoid brushing at the surgical site during the same period until the sutures were completely resorbed. Finally, Augmentin 875/125 mg (2 times a day for 1 week) and Ibuprofen 600 mg (2 times a day for 5 days) were also prescribed.

Recalls were scheduled at 2 weeks, and 1, 2, 3, and 4 months post op.

### 2.3. Stage 2

**Figure 11:**
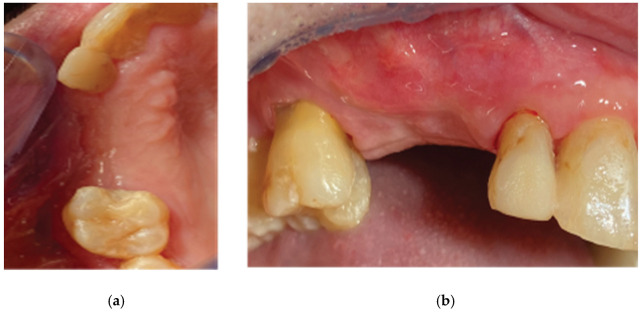
Clinical situation at 3 months post op—(**a**) occlusal view, (**b**) lateral view.

**Figure 12:**
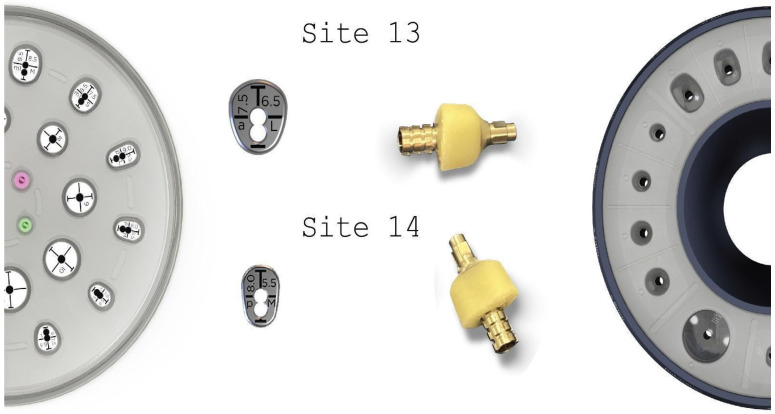
Generation of customized abutments with the VPI Cervico System.

Three months after the first surgery and an uneventful healing, the alveolar ridge showed an evident increase in its dimensions compared to the pre-op clinical situation (Figure 11). It was considered safe to remove the healing abutments from the osseointegrated SLA Straumann implants and replace them with anatomical abutments for cervical profile generation. In order to optimize the soft tissue, we used the VPI Cervico system for the chairside fabrication of the 2 anatomical profiles (AL profile for implant 13 and PM for implant 14) mounted on 2 engaged provisional cylinders for regular platform BLT implants (Straumann Group, Basel, Switzerland) and nanohybrid flow composite (Vertise Flow, Kerr—KaVo Kerr, Brea, CA USA) (Figure 12).

**Figure 13:**
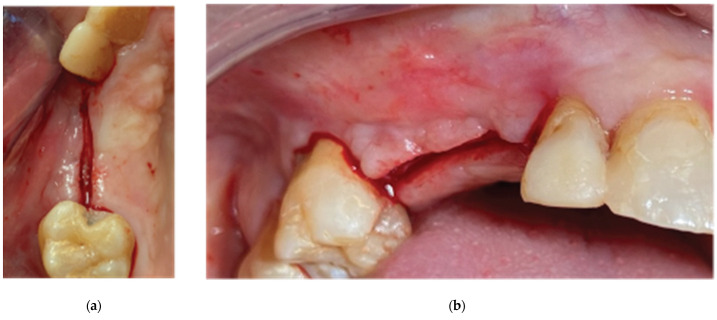
Partial-thickness incision—(**a**) occlusal view, (**b**) lateral view.

**Figure 14:**
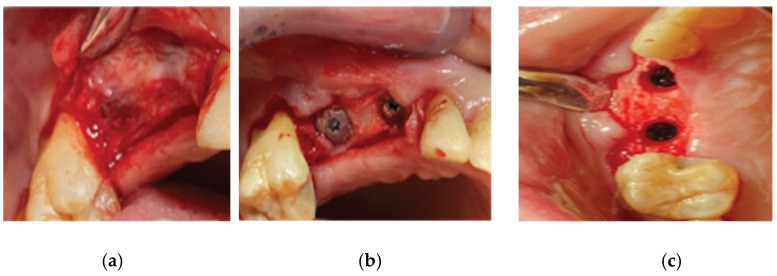
Partial-thickness flap and uncovering of the healing abutments’ platform. (**a**) clinical situation after partial thickness flap elevation, (**b**) uncovering the healing abutments, (**c**) clinical situation after removing the healing abutments.

Following customized abutment generation, a split-thickness preparation was initiated with the help of a 15 blade, starting at a depth of 2 mm from the marginal soft tissue level to avoid any interference with the not-yet-maturated grafted material (Lamina barrier and particulate graft) below (Figure 13). Further extension of the splitting process with the blade and the assistance of a periosteal elevator reached a level just below the mucogingival line on the buccal side and 5 mm apically of the soft tissue margin on the palatal side. Two split-thickness flaps (buccal and palatal) were raised, the 2 healing abutments were removed, and a 6 mm supracrestal complex height from the implant platform to the marginal tissue edge was evaluated with a periodontal probe (PCPUNC156 Hu-Friedy, HuFriedyGroup, Chicago, IL USA) for both site 13 and site 14 (Figure 14) [21].

**Figure 15:**
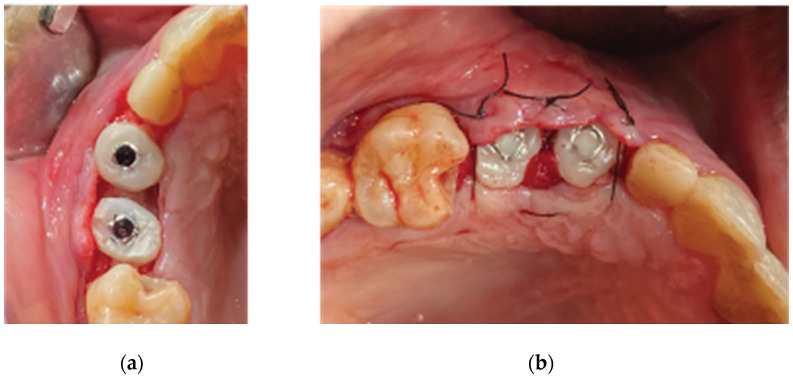
Stage 2 procedure. (**a**) replacement of the healing abutments with VPI Cervico abutments, (**b**) flap closure via adding collagen sponges within the inter-proximal areas.

The 2 anatomical VPI Cervico abutments were placed and secured over the implants after their surface was disinfected with alcohol and cleaned with a steamer. The 2 flaps were then secured around the abutments with 5.0 silk interrupted sutures and compressed trimmed pure collagen (Spongostan—Ethicon Inc, Somerville, NJ, USA) was used in the interproximal areas to fill the spaces between the marginal edges of the flaps (Figure 15). The same post-surgical instructions as in stage 1 of the procedure were given to the patient, and Azithromycin 500 mgr was prescribed (1 g single same-day dose) to enhance soft tissue healing.

### 2.4. Case 2

**Figure 16:**
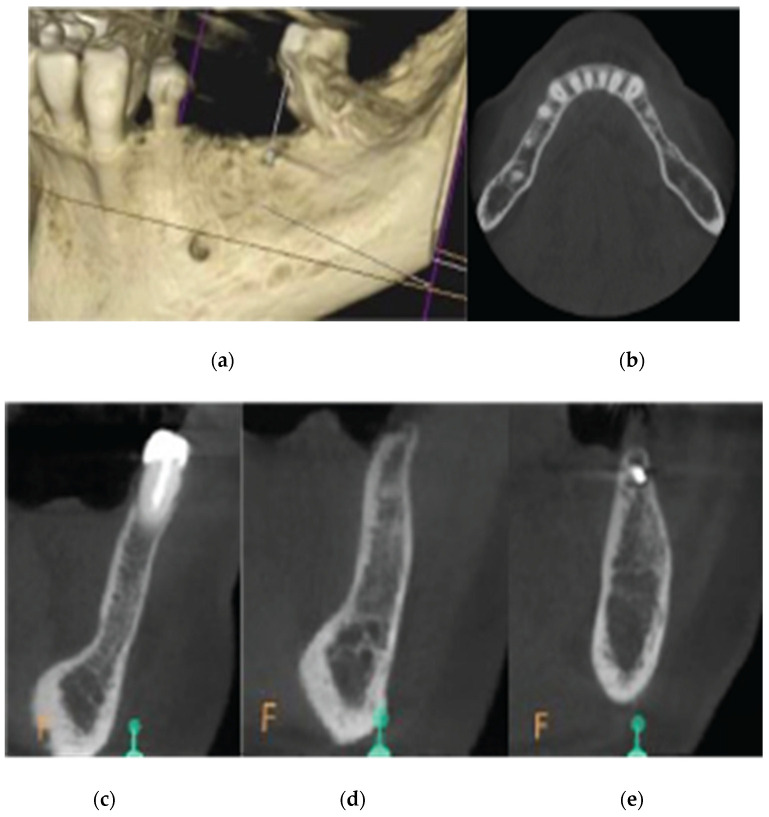
Pre-op CBCT situation—(**a**) 3D representation of the defect, (**b**) axial mandibular image. (**c**) Cross-sectional images of site 34, (**d**) site 35, and (**e**) site 36.

A 68-year-old female patient attended our clinic and requested a fixed solution for the posterior edentulous 35–36 area of quadrant 3. The patient was a healthy non-smoker without active periodontal disease (no attachment loss, pockets on probing, and BoP). A pre-op cone beam computed tomography (CBCT) scan was performed on the lower third quadrant. The results revealed an important horizontal atrophy of the ridge at the area from the distal aspect of tooth element 34 to the mesial aspect of wisdom tooth 38. Bone density was defined as type 3 [15] and the distance from the most coronal edge of the alveolar ridge to the inferior alveolar canal was enough to place dental implants (more than 10 mm). Residual endodontic filling material within the bone structure was identified at area 36. A periapical lesion apically of 38 roots with underfilled root canal treatment and bone loss at the furcation area were also noted (Figure 16).

**Figure 17:**
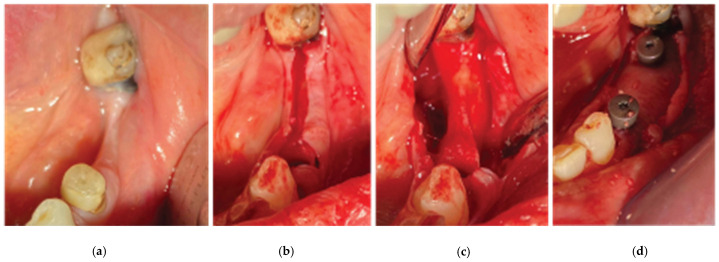
Stage 1 of the surgical procedure—(**a**) initial situation, (**b**) incision design and extraction of tooth 34, (**c**) thin alveolar ridge after flap elevation, (**d**) healing abutment–Lamina compound following overgrafting.

The clinical examination revealed a keratinized tissue width of 4 mm upon a thin alveolar ridge, a mesioversion of element 38 that additionally presented a type 2 mobility, and a narrow class 3 furcation (Figure 17).

We decided that the short-rooted element 34 should be extracted and replaced by a dental implant immediately after extraction, along with a Poncho Lamina accelerated protocol for both implant site 34 and 36. As tooth 27 was missing, and the patient did not want to replace it, an implant rehabilitation for a future 3-unit bridge 34–36 was selected as the best treatment option. Tooth 38 was left to be extracted at stage 2 of the surgical procedure. In this particular case, we used the same material as in case 1 but we added Everstick glass fibers to fabricate a provisional 3-unit fixture for cervical profile generation at 34–36 during stage 2 of the procedure.

### 2.5. Stage 1

Following the administering of local anesthesia (Articaine Hydrochloride/Adrenaline), the same surgical workflow was followed as in case 1: after flap elevation and release on both sides of the ridge [22], tooth element 34 was extracted and 2 Straumann SLA BLT 4.1-diameter implants (10 mm length for the 34 implant, and 8 mm length for the 36) were placed subcrestally with the Versah expansion protocol at a primary stability of 35 N.cm. The site was overgrafted with 2 g of Genos 0.5 g (particle size: 0.5–1 mm), and the trimmed Curved Cortical Lamina was secured above the implant platforms with healing abutments of 5 mm diameter and 6 mm height (StraumannGroup, Basel, Switzerland) (Figure 17). Flap closure was achieved around the abutments with the 2 suture layers and the same suturing material described in stage 1, and muscle pulling reduction was performed with a superficial incision at the depth of the vestibule, as previously noted.

Post-surgical instructions and medication were identical to those in Case 1, and the patient was advised to avoid chewing hard-consistency food in the left side of the mouth cavity.

### 2.6. Stage 2

**Figure 18:**
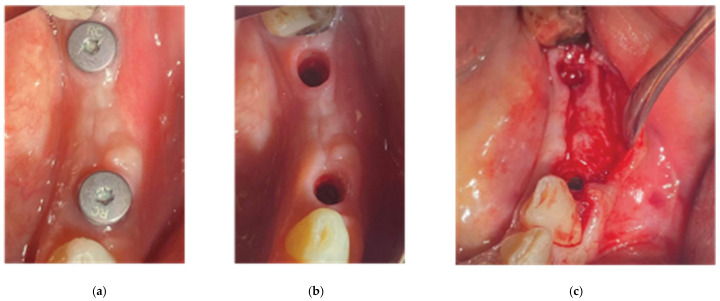
Stage 2 of the surgical procedure—(**a**) initial situation, (**b**) emergence profiles after stock abutment removal, (**c**) split-thickness preparation.

**Figure 19:**
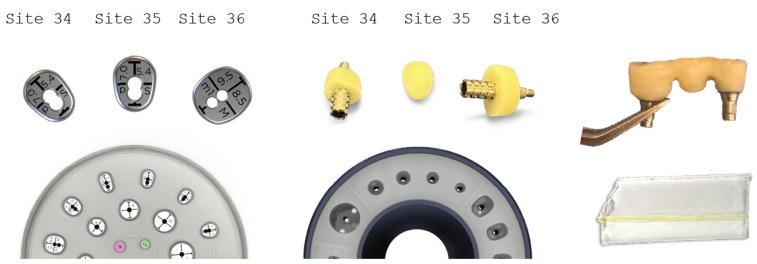
Generation of a3-profile fixture with VPI Cervico System and Periostick glass fibers.

Two months after the first surgery, the Straumann healing abutments were removed from the SLA osseointegrated implants. The healing screws were replaced with VPI Cervico anatomical abutments, using the surgical steps corresponding with those in the same stage of case 1 (Figure 18). However, in this particular case, a 3-unit fixture was generated to prepare the site for a future 34–36 bridge. Two anatomical abutments, 34 and 36, were fabricated using non-engaged Straumann provisional cylinders (Straumann Group, Basel, Switzerland) and a nanohybrid flow composite (Vertise Flow, Kerr—KaVo Kerr, CA, USA). The pontic element’s generation was based on the Cervical Socket Plug technique protocol (Figure 19) [23]. Using cylindrical diamond burs at high speed, 2 grooves (one buccal and one lingual) were made on all 3 units at their most coronal edge. After abutments 34 and 36 were secured over the implant platforms, and the pontic profile unit was correctly positioned, the nanohybrid composite flow filled the coronal area of the interproximal spaces in order to connect the 3 profiles. The connection was furthermore enhanced with the placement of everStickPERIO glass ionomer fibers (GC Europe N.V.) in the groove area of the 3 units, covered with flow composite. The fixture was then removed from the surgical site and polished, and composite was carefully trimmed out from the apical areas of the interproximal spaces to assure interdental cleaning (Figure 19).

**Figure 20:**
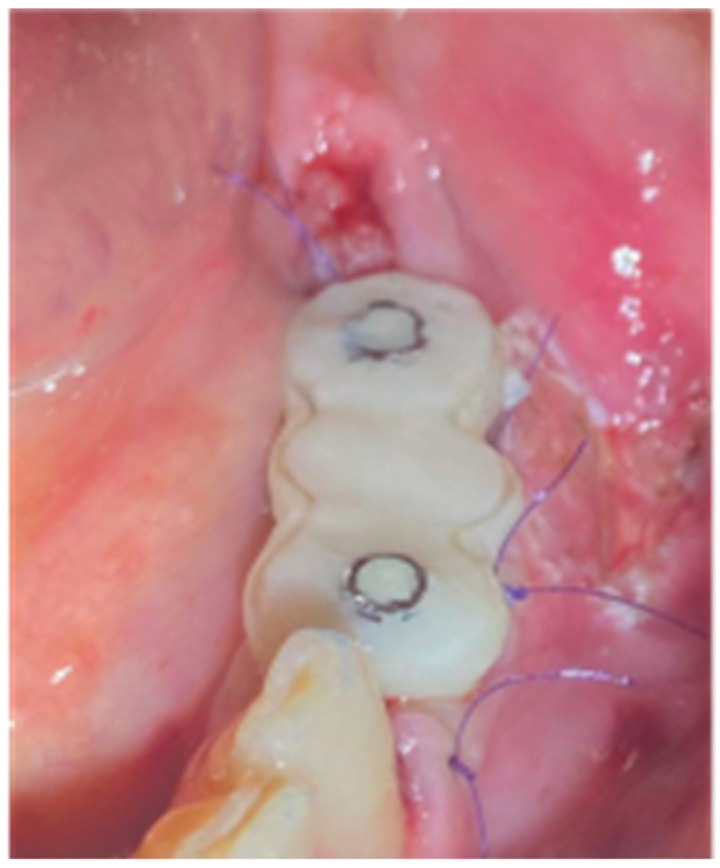
The 3-unit profile fixture in situ and flap closure.

After the sutures were placed, we used a diode laser device (Smart M Pro, Lasotronix, Piaseczno, Poland) with a 980 nm optic fiber activated for photoablation at 2.5 W for deepening the vestibule at the area buccally of the pontic site and site 36 (Figure 20). Finally, the occlusion was carefully corrected to avoid any contact of the profiles with the antagonists, and post-operative instructions and medication were prescribed as in case 1.

A diode laser was used to reduce muscle pulling.

### 2.7. Data Collection

**Figure 21:**
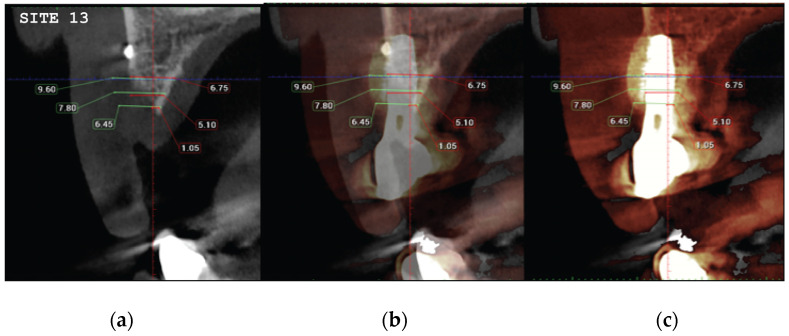

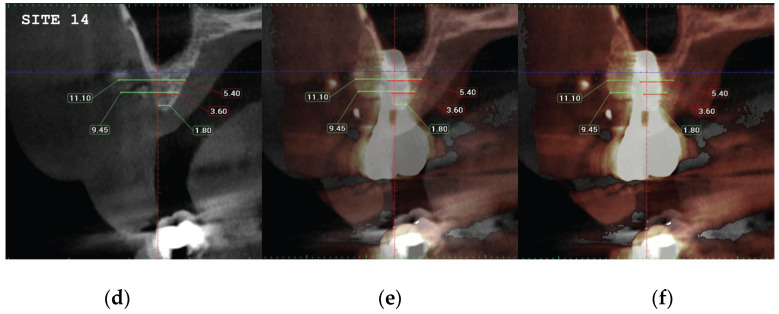
(**a**) The T1 CBCT image of site 13, (**b**) the superposed images T1–T2, (**c**) and the post-image T2, with measurements overlaid. (**d**–**f**) Similar setup for the site of premolar 14.

**Figure 22:**
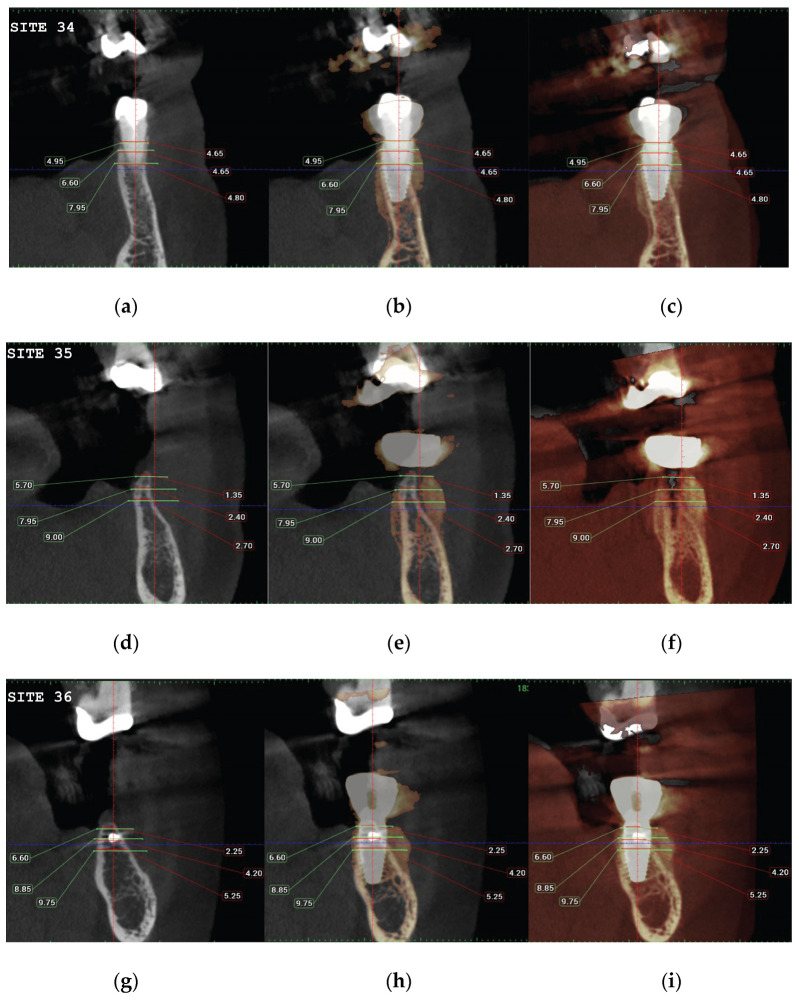
(**a**) The T1 CBCT image of site 34, (**b**) the superposed images T1-T2, (**c**) and the post-image T2, with measurements overlaid. (**d**) The T1 CBCT image of site 35, (**e**) the superposed images T1-T2, (**f**) and the post-image T2, with measurements overlaid. (**g**–**i**) Similar setup for the molar 36 site.

**Figure 23:**
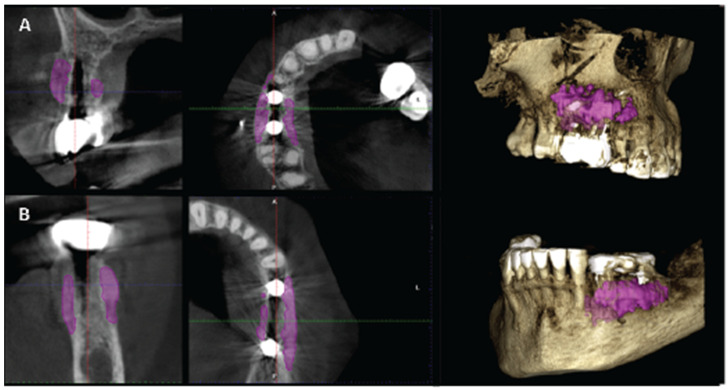
(**A**) (case 1) and (**B**) (case 2) are the T2 images of both patients with manually segmented bone volume gain.

CBCT images were obtained using a CS8100 3D Carestream computed radiography system (Carestream Dental LLC, Atlanta, GA USA) at 0.150 mm voxel size and a field of view of 8 × 9 cm prior (T1) and 16 weeks (T2) after implant placement. The original files in DICOM format were imported into the Romexis software (Version 6.0.1.812, Planmeca Oy, Tuusula, Finland) for analysis. Both the T1 and T2 datasets were superimposed in the software, using a point-based registration and, if needed, further manual alignment by an experienced oral imaging specialist. Then, 2D measurements were acquired on both registered volumes, with a slider tool helping to hide and show the second superposed volume. Measurements were carried out first on T1, and then on T2. The cross-section in the middle of the site on the axial slice and perpendicular to the alveolar crest was used to measure the alveolar widths at 1 mm, 3 mm, and 5 mm heights (Figure 21 and Figure 22). An approximate bone volume segmentation of the bone gain was performed on both volumes using a manual segmentation tool to visualize the gain in 3D (see Figure 23).

## 3. Results

Clinical evaluation for soft tissue and CBCT data analysis for hard tissue configuration were performed for both cases at T1—pre-op and T2—4 months post-op. 

### Soft Tissue Configuration Results:

**Figure 24:**
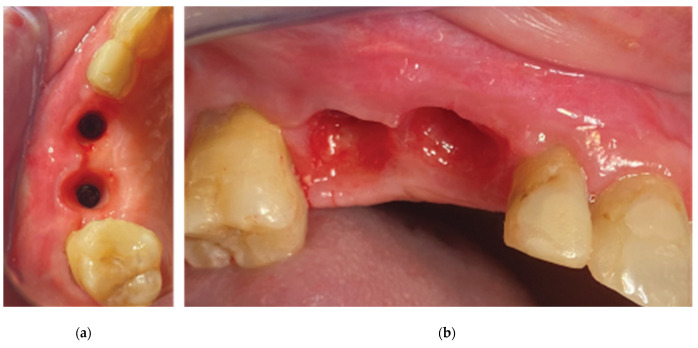
Case 1—final clinical result: (**a**) occlusal view, (**b**) lateral view.

**Figure 25:**
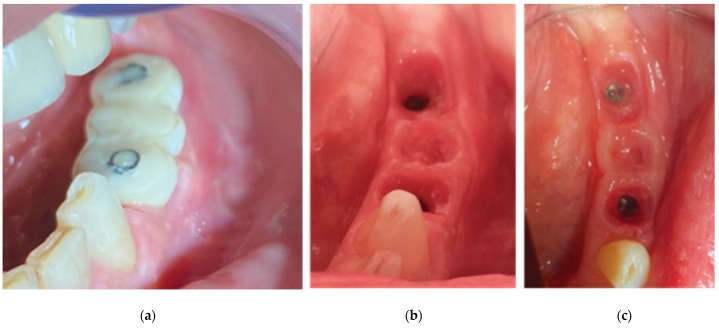
The final clinical situation before (**a**) and after removing the three-profile-unit fixture (**b**,**c**).

Both cases revealed adequate soft tissue configuration with “mimicking the nature” cervical profiles after the removal of the VPI Cercico abutments at T2 (Figure 24 and Figure 25).

**Figure 26:**
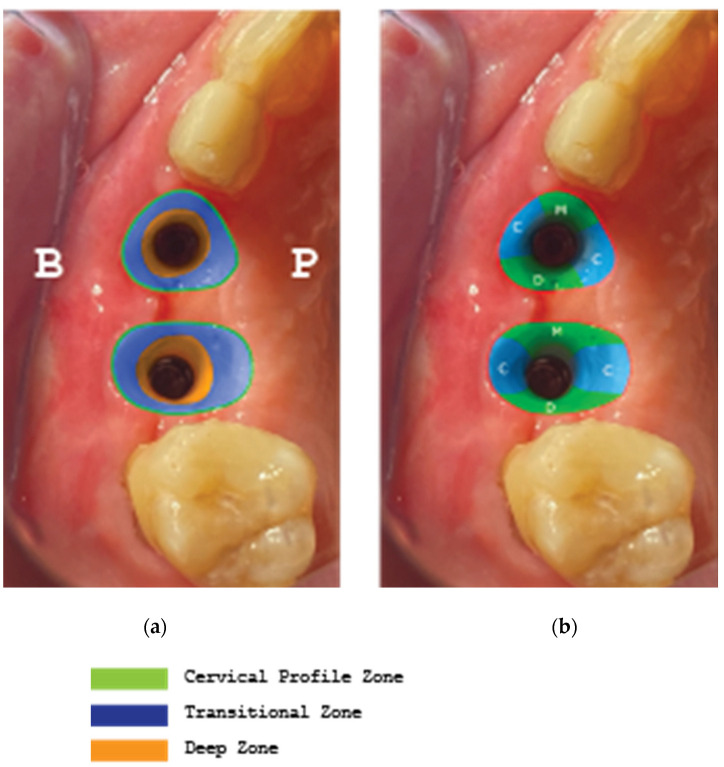
(**a**) Supracrestal complex configuration for case 1, (**b**) Height measurements at six perimetric areas: three buccal-B (mesial-MB, central-CB, distal-DB) and three palatal-P (mesial-MP, central-CP, distal-DP).

**Figure 27:**
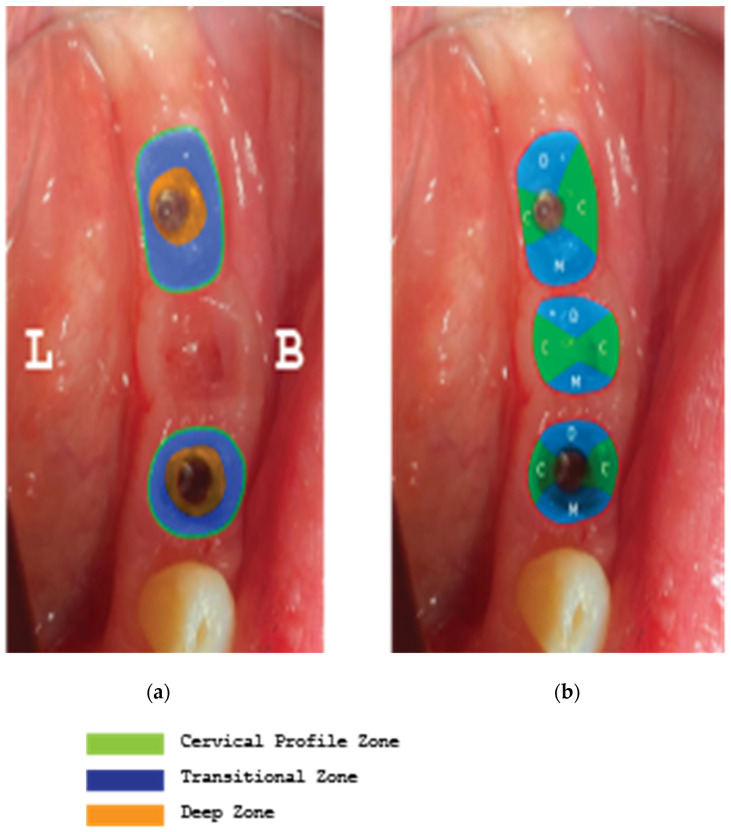
(**a**) Supracrestal complex configuration for case 2, (**b**) Height measurements at six perimetric areas: three buccal-B (mesial-MB, central-CB, distal-DB) and three lingual-L (mesial-ML, central-CL, distal-DL).

The implant supracrestal complex height was recorded with the help of a periodontal probe (PCPUNC156 Hu-Friedy, HuFriedyGroup, Chicago, IL, USA) for all upper and lower sites at Tx (stage 2) and T2 (Figure 26 and Figure 27).

Regarding the soft tissue configuration, results were evaluated at a mm scale taken at six perimetric (mesio-central–distal) points both on the buccal and the palatal/lingual sides. The height was evaluated from the implant platform to the marginal soft tissue edge (Table 1).

Regarding the Hard tissue configuration, results were evaluated as alveolar width measurements at 1, 3, and 5 mm heights in mm. The measurments were taken at T1 pre-op and T2 post-op. All T2 values were increased compared to T1 at all three alveolar heights (Table 2).

## 4. Discussion

The present article features a novel biphasic accelerated protocol that enables the surgeon to perform, at partial edentulous sites, implant treatment and both soft and hard tissue augmentation in just 4 months.

**Figure 28:**
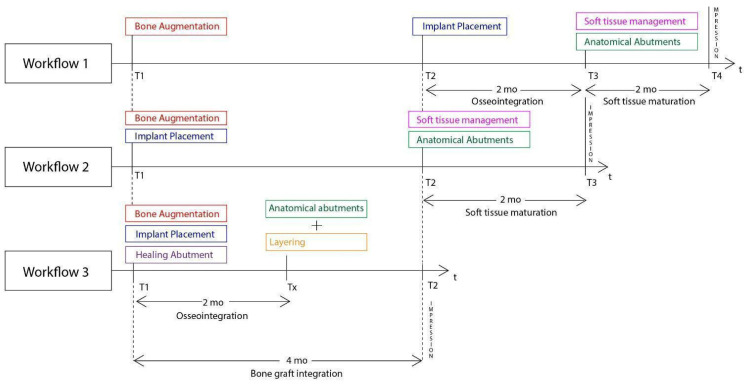
Superposition of three Workflow charts for bone augmentation procedures with Straumann SLA implants: Workflow 1: Usual ridge augmentation protocol with delayed implant placement; Workflow 2: Augmentation staged protocol with simultaneous implant placement; Workflow 3: Accelerated Poncho Lamina protocol.

In general, similar workflows, especially in the case of 3D defects, require three surgical steps: during the first, bone augmentation is performed with various techniques and, only 4–6 months later, the implants may be placed after bone graft maturation. Following implant osseointegration at stage 2, which normally takes 3 months in the mandible and 4 months in the upper maxilla, the final stage 3 for soft tissue management is initiated. In most cases, such management requires soft tissue grafting procedures (connective and free gingival grafts) from various donor sites (hard palate, tuberosity). Finally, 1–2 months after soft tissue maturation, an optimized result with cervical profile generation is achieved by means of the provisional fixed prosthesis or customized abutments that are used during stage 3. Overall, we may conclude that, during such processes, the treatment takes at least 6 months. Even if the surgeon decides to combine the first two stages and place the implants simultaneously, while augmenting the alveolar ridge, an optimal hard and soft tissue result will require 5–6 months (Figure 28). Taking in account the multiple interventions and the need for hard/soft tissue grafting with additional surgical acts at donor sites, the usual workflow may be both long and painful for the patient and include non-negligible post-operative risks, such as post-operative bleeding (palatal donor sites), necrosis in intraoral hard tissue donor sites (e.g., the chin and ramus areas), and site infection while using non-resorbable materials (titanium and PTFE meshes, tenting screws, and pins) after exposure [24,25]. In this context, every single intervention induces scar tissue formation that leads to more difficult tissue manipulation in the following stage.

**Figure 29:**
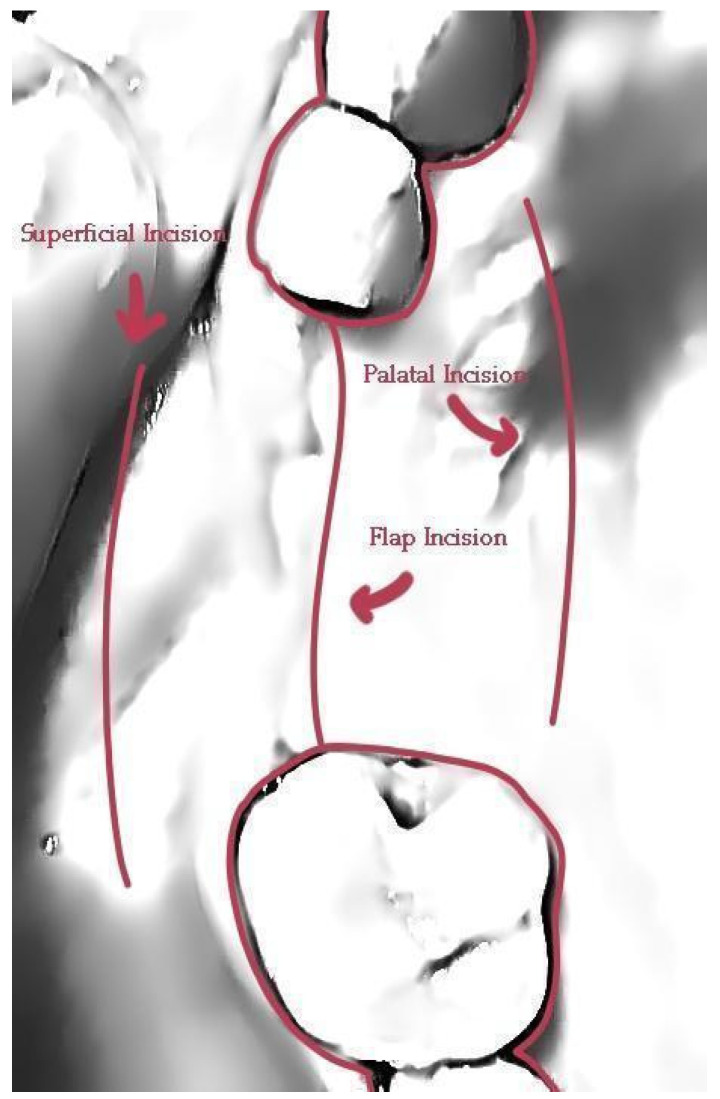
The incisions used to ensure primary closure above the stock abutments for case 1.

**Figure 30:**
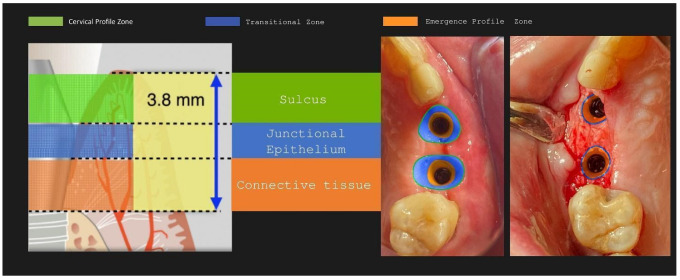
Implant supracrestal complex and partial-thickness preparation ([27] modified).

**Figure 31:**
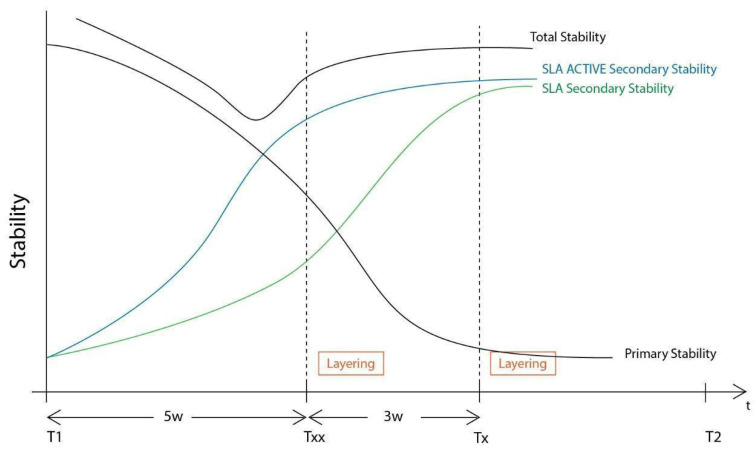
The differences in the layering procedure initiation between the Poncho Lamina accelerated protocol when using SLA Active implants (Txx) compared to SLA (Tx).

Compared to the above, the Poncho Lamina accelerated protocol is based on a completely different strategy that features two main elements:

1. A combined implant placement and bone/soft tissue augmentation with the use of the cortical lamina and the healing abutment compound during stage 1.As well as the importance of theabutment–lamina compound, some other features should be noted:
  - A good primary stability in the implants is primarily achieved with the implementation of osseodensification burs when bone density type is 3 or 4. Versah drills also provide native bone preservation through ridge expansion, limit the risk of bone dehiscences during drilling, provide the possibility of adding a simultaneous crestal protocol 1 or 2 sinus lift, and improve osseointegration by reducing the direct contact of xenograft particles with the implant surface whenever such grafting materials are used [26].
  - Overgrafting the site supplies the necessary volume for both hard and soft tissue augmentation. This avoids the need for subsequent soft tissue augmentation and additional interventions at donor sites in stage 2. The use of healing abutments over the implants add the foundations of emergence profile generation very early in the treatment, and these will later be optimized in the following stage.
  - The occlusal connection between the lamina cortical barrier and the healing abutments provides a very precise and sufficient stabilization that prevents the use of additional fixation elements and the need to remove them at a later stage (e.g., screws or pins).
  - Horizontal mattress sutures further stabilize the barrier at a lower level over the grafting granulated material on both the buccal and palatal/lingual sides of the site. The consistency and elasticity of the cortical lamina creates a firm structure that both protects the overgrafted material and ensures tenting support for the soft tissues.
  - The use of a specific incision design without vertical releases for further envelope flap elevation preserves the vascular integrity within the surgical site and the tissue in the interproximal areas of neighboring tooth elements through partial-thickness preparation. This meticulous, minimally invasive site preparation is mandatory to ensure bone graft integration and less soft tissue retraction during the healing period.
  - The correct adaptation of the soft tissue around (case 2) or over the abutments (case 1 with primary closure) during flap closure is achieved with sutures placed in different layers. This enables a better distribution and absorption of the muscle pull force on the soft tissues that cover the barrier and reduces the risk of lamina exposure. Uncovered areas over the cortical sheet would lead to resorption and soft tissue apical migration. Further use of a superficial incision deep in the vestibule concentrates the muscle pull force in an area away from the flap marginal edges, while palatal incisions parallel to the alveolar ridge in upper maxilla cases provide elasticity in the tissue for hermetic closure over the barrier (Figure 29).
2. The setting and initiation of the second stage occur earlier in the timeframe, through ”a window of opportunity” created by the osseointegration rate (Tx, see Figure 28). When the implant secondary stability is adequate for the removal of the healing abutments, a layering partial-thickness preparation takes place, and the anatomical abutments are used for cervical profile generation. However, it is important to highlight some details concerning this soft tissue management stage:
  - The layering preparation is performed with partial-thickness flaps to avoid any interference and trauma to any barrier remnants and granulated material below. The incision is located within the transitional zone and above the deep zone of the supracrestal complex (Figure 30) [27]. Any incision below that level may induce trauma to the vascular complex and rigid collagen type 1 fibers that provide tissue stability over the implant platform. This stability protects and preserves the integrity of the coronal part of the grafting material during its maturation and prevents further resorption [28,29]. Although there are articles showing that partial-thickness flaps also lead to bone loss, it is widely accepted, in general, that such surgical acts result in faster healing, less post-operative pain, and the protection of fragile structures in deeper layers of the site [30].
  - After the removal and replacement of the healing abutments, adaptation and marginal soft tissue flap edge closure could be better achieved with an apical reposition flap procedure rather than an hermetic closure around the keratinized abutments for cervical profile generation. This ensures keratinized tissue preservation and reduces the need for free gingival grafts, which may still be used during this stage but are more invasive. Interproximal areas may often remain uncovered after flap closure, especially in upper maxilla cases, but the use of compressed collagen trimmed sponges provides the support for epithelial migration over their surface to promote papilla formation. This happens often in upper maxilla cases like the one demonstrated in this case report.
  - The precise moment of stage 2 initiation may vary and is dependent on the implant osseointegration rate. In reality, the use of implants with novel and more hydrophilic surface coatings may trigger the layering process and abutment replacement earlier in the treatment (Figure 31) [31,32].

In this case report, we decided, along with the full protocol description, to present two particularly interesting cases.

Case 1 features the placement of two adjacent implants in the upper maxilla, where a 3D defect is present. Along with the horizontal and vertical hard and soft tissue augmentation, a Versah protocol 1 crestal sinus lift was also added to the whole treatment. Attention was paid to the augmentation itself and the protection of the implants during the osseointegration period, in a region where keratinized tissue was sufficient. This is the main reason why primary closure over the healing abutments was selected at the end of stage 1.

Case 2 demonstrates a more demanding situation regarding soft tissue management, as it required cervical profile generation for a future 3-unit bridge with pontic site development in an area where keratinized tissue is often scarce. Here, instead of suturing the soft tissue above the abutments, the latter remained partially uncovered with the limited keratinized tissue secured around them. A full coverage of the abutment platforms would raise the risk of their early exposure through thin soft tissue and keratinized tissue loss.

In both cases, attached tissue was still present at the end of soft tissue maturation around the implant sites, but we strongly advise performing an apical repositioning of the buccal flaps for upper maxilla cases rather than proceeding to a simple tissue adaptation around the customized abutments and free gingival grafts, if needed, at stage 2 of the procedure for mandibular cases.

The supracrestal heights were recorded at Tx and T2 for both cases. The measurements at T2 were at least 4 mm for the mandibular sites and reached bigger values at the maxillary one, due to a higher initial soft tissue thickness and primary closure over the abutment platforms at the end of stage 1. A 1 mm loss was recorded when the measurements were compared between Tx and T2. This height reduction conforms to other findings in the bibliography, especially concerning anterior sites when healing abutments are to be replaced with anatomical or provisional crowns [33]. This is to be taken into consideration when positioning the implants at a subcrestal level during stage 1 of the procedure.

Despite the fact that the soft tissue data measurements report values above the necessary 4 mm limit for an adequate and sufficient supracrestal implant complex height and cervical profile generation were achieved in all five upper and lower sites, STL files with superposition and volumetric analysis will be needed in future studies to evaluate the soft tissue gains at T2.

Significant gains in bone configuration were recorded via CBCT data collection and alveolar width measurements at three different levels of the ridge for both the T1 and the T2 dataset. The values at T2 for the two upper maxilla sites ranged from a minimum of 6.45 mm to a maximum of 11.10 mm, with the highest difference from T2 to T1 recorded at the distal 14 site. The lower site values ranged from a minimum of 4.95 mm to a maximum of 9.75 mm, with the highest difference from T2 to T1 at pontic site 35. Although these values were taken at different levels, if we consider that at least 1–2 mm of surrounding hard tissue are necessary for long-term stability around dental implants, the current findings show an exceptional hard tissue volume around all four implant sites [34,35]. In particular, the value and importance of having a firm attachment and stabilization of the cortical barrier within the healing abutment platforms and over the overgrafted granulated material, at a higher level than that defined from the residual bony ridge at T1,is clearly demonstrated in the cross-sectional CBCT images of pontic site 35. Finally, we may appreciate an overall 3D bone volume gain visualization at T2 after bone segmentation volume gains, which confirms the results in the cross-sectional images. Future studies could also include volumetric analysis on bone gains through STL files.

**Figure 32:**
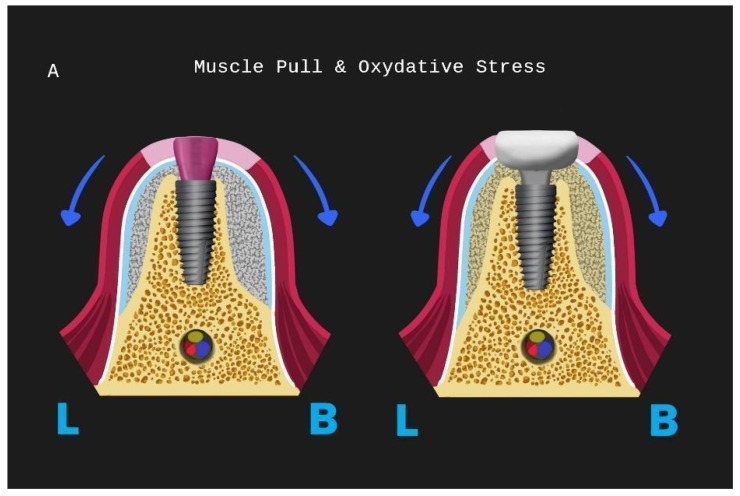

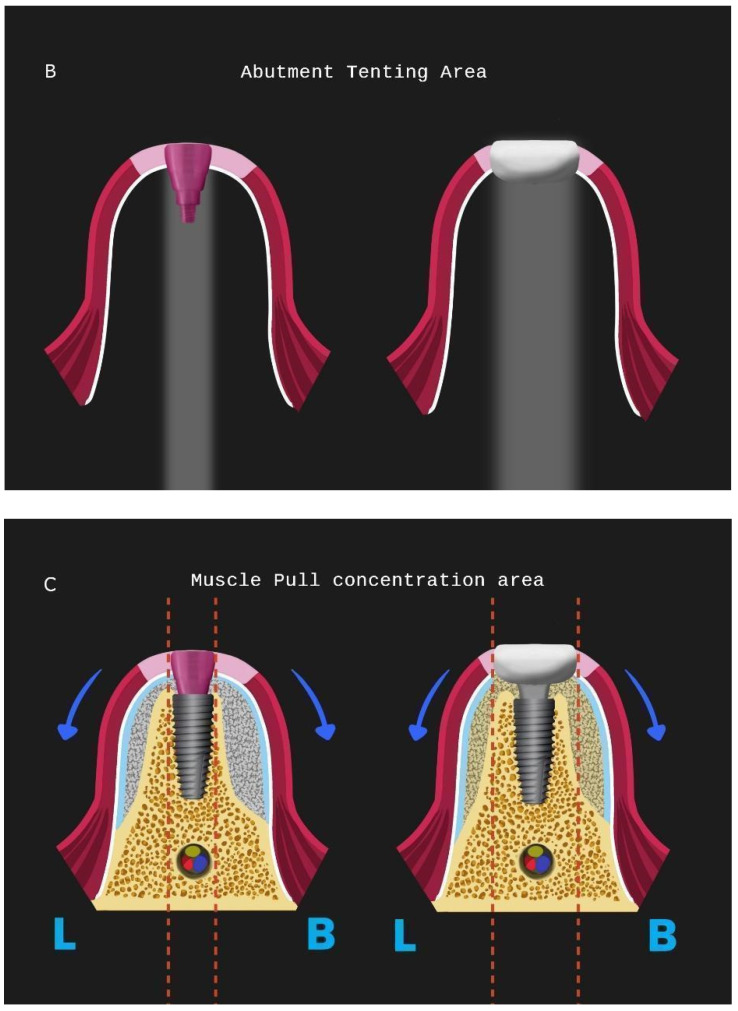
The Poncho Lamina accelerated protocol and oxidative stress for a lower implant site: (**A**) muscle pull direction for stages 1 and 2, (**B**) tenting area below healing and customised abutment, (**C**) muscle pull concentration area for stages 1 and 2 (L is lingual, B is buccal).

Following the above-mentioned points, the accelerated protocol with the Poncho Lamina technique presents advantages over the usual longer protocols already established in the bibliography and the clinical practice. It is clear that, in this particular workflow, the overall treatment duration is reduced through the correct use of its two basic elements, already described in this section. Nevertheless, the T1–T2 time length is dependent on the bone-grafting material integration alone (Figure 28). This clearly suggests that, in the future, novel bone regeneration grafting materials could further reduce the overall treatment duration to below the 4-month limit. In our opinion, in this particular process, the importance of reducing the treatment time does not only lie in providing a faster treatment for the patient. It is, rather, essential to oxidative stress management during the healing period: as the window of opportunity at Tx with the initiation of the layering process moves closer to the T1 timeframe, the use of anatomical abutments generated with the VPI Cervico system for a longer period until T2 shifts the muscle pull concentration region and provides a larger tenting area for the non-maturated grafting material below [36,37]. This might finally be of a greater importance than the treatment duration itself, as it may have a great impact on the soft and hard tissue augmentation results at T2. In this context, early soft tissue optimization management would provide the ideal conditions for overall hard tissue integration and configuration (Figure 32).

## 5. Conclusions

This article introduces a modern approach to simultaneous implant placement and bone/soft tissue optimization in adjacent implant sites, with the use of a cortical barrier mounted on healing abutments replaced at a second early stage by customized abutments generated with a chairside system. This novel workflow offers the following:

− a faster alternative treatment for the patient due to a significantly reduced duration compared with other approaches;
− less invasive than similar augmentation procedures in need of donor sites for autologous grafting;
− uses only resorbable grafting material and reduces scar tissue formation;
− less painful for the patient and reduces the risk of post-operative complications.

Two cases are reported, showing significant gains in both hard and soft tissue volume and configuration 4 months after the beginning of the treatment. Further prospective case series studies with longer recall data collection are needed in the future to evaluate the efficiency and validity of the presented protocol.

## Figures and Tables

**Table 1:** Results at a mm scale taken at six perimetric (mesio-central–distal) points both on the buccal and the palatal/lingual sides. The height was evaluated from the implant platform to the marginal soft tissue edge.

Element		Site	Value (mm) Tx	Value (mm) T2
**13**	Buccal	M	6	5
		C	6	5
		D	6	5
	Palatal	M	6	5
		C	6	5
		D	6	5
**14**	Buccal	M	6	5
		C	6	5
		D	6	5
	Palatal	M	6	5
		C	6	5
		D	6	5
**34**	Buccal	M	5	4
		C	5	4
		D	5	4
	Lingual	M	5	4
		C	5	4
		D	5	4
**35**	Buccal	M	0	3
		C	0	3
		D	0	3
	Lingual	M	0	3
		C	0	3
		D	0	3
**36**	Buccal	M	6	4
		C	6	4
		D	6	4
	Lingual	M	6	4
		C	6	4
		D	6	4

**Table 2:** The alveolar width measurements at 1, 3, and 5 mm heights in mm. The measurements were taken at T1 pre-op and T2 post-op. All T2 values were increased compared to T1 at all three alveolar heights.

Site	Level	PRE (T1)	POST (T2)
**CASE1-13**	1 mm	1.50	6.45
	3 mm	5.10	7.80
	5 mm	6.75	9.60
**CASE1-14**	1 mm	0	1.80
	3 mm	3.60	9.45
	5 mm	5.40	11.10
**CASE2-34**	1 mm	4.65	4.95
	3 mm	4.65	6.60
	5 mm	4.80	7.95
**CASE2-35**	1 mm	1.35	5.70
	3 mm	2.40	7.95
	5 mm	2.70	9.00
**CASE2-36**	1 mm	2.25	6.60
	3 mm	4.20	8.85
	5 mm	5.25	9.75

## Data Availability

Data are contained within the article.
